# Evasion of the Immune Response by *Trypanosoma cruzi* during Acute Infection

**DOI:** 10.3389/fimmu.2015.00659

**Published:** 2016-01-18

**Authors:** Mariana S. Cardoso, João Luís Reis-Cunha, Daniella C. Bartholomeu

**Affiliations:** ^1^Laboratório de Imunologia e Genômica de Parasitos, Departamento de Parasitologia, Universidade Federal de Minas Gerais, Belo Horizonte, Minas Gerais, Brazil

**Keywords:** *T. cruzi* acute infection, immune response, *T. cruzi* immune evasion, Chagas disease, immunomodulation

## Abstract

*Trypanosoma cruzi* is the etiologic agent of Chagas disease, a neglected tropical disease that affects millions of people mainly in Latin America. To establish a life-long infection, *T. cruzi* must subvert the vertebrate host’s immune system, using strategies that can be traced to the parasite’s life cycle. Once inside the vertebrate host, metacyclic trypomastigotes rapidly invade a wide variety of nucleated host cells in a membrane-bound compartment known as the parasitophorous vacuole, which fuses to lysosomes, originating the phagolysosome. In this compartment, the parasite relies on a complex network of antioxidant enzymes to shield itself from lysosomal oxygen and nitrogen reactive species. Lysosomal acidification of the parasitophorous vacuole is an important factor that allows trypomastigote escape from the extremely oxidative environment of the phagolysosome to the cytoplasm, where it differentiates into amastigote forms. In the cytosol of infected macrophages, oxidative stress instead of being detrimental to the parasite, favors amastigote burden, which then differentiates into bloodstream trypomastigotes. Trypomastigotes released in the bloodstream upon the rupture of the host cell membrane express surface molecules, such as calreticulin and GP160 proteins, which disrupt initial and key components of the complement pathway, while others such as glycosylphosphatidylinositol-mucins stimulate immunoregulatory receptors, delaying the progression of a protective immune response. After an immunologically silent entry at the early phase of infection, *T. cruzi* elicits polyclonal B cell activation, hypergammaglobulinemia, and unspecific anti-*T. cruzi* antibodies, which are inefficient in controlling the infection. Additionally, the coexpression of several related, but not identical, epitopes derived from trypomastigote surface proteins delays the generation of *T. cruzi*-specific neutralizing antibodies. Later in the infection, the establishment of an anti-*T. cruzi* CD8^+^ immune response focused on the parasite’s immunodominant epitopes controls parasitemia and tissue infection, but fails to completely eliminate the parasite. This outcome is not detrimental to the parasite, as it reduces host mortality and maintains the parasite infectivity toward the insect vectors.

## Introduction

Chagas disease, also known as American trypanosomiasis, is caused by the protozoan parasite *Trypanosoma cruzi*, a highly diverse taxon. This disease is endemic to Latin America, with sporadic cases mainly in the United States and Europe, and affects nearly 8 million people, accounting for the loss of 662,000 disability-adjusted life years (1–3) (WHO).^1^ This parasite alternates between invertebrate hematophagous insects from the Reduviidae family and a broad range of mammalian hosts (4). Although the estimated period for *T. cruzi* speciation is still a matter of debate (5–9), recent molecular studies suggest that the ancestor of *T. cruzi* may have been introduced to South America approximately 7–10 million years ago (8, 9), and the oldest record of human infection dates from 9,000 years ago (10). Since then, this parasite has evolved fascinating strategies to evade and subvert the mammalian host immune system, leading to life-long last infections. These strategies can be traced to the parasite’s life cycle.

*Trypanosoma cruzi* metacyclic trypomastigotes are released in the feces or urine of the triatomine vector after a blood meal. These forms are able to infect the mammalian host if they encounter mucosa or discontinuous regions in the epithelium. Once inside the host, the parasite rapidly infects a wide variety of nucleated mammalian cells (11–13). *T. cruzi* relies on an arsenal of polymorphic glycosylphosphatidylinositol (GPI)-anchored surface proteins, such as *trans*-sialidases, mucins, and others, to attach and invade host cells, leading to the formation of the parasitophorous vacuole (14, 15). After lysosomes are fused to the parasitophorous vacuole, parasite survival is mediated by a complex network of antioxidant enzymes, such as peroxidases and superoxide dismutases (SODs), that shield it from reactive oxygen and nitrogen species (16). In fact, instead of being detrimental, the lysosomal acidification is an important signal for activating key mechanisms that allow the parasite to escape from the phagosome into the cytoplasm, where it differentiates into the replicative amastigote forms. After several rounds of duplication, the amastigotes differentiate into infective bloodstream trypomastigotes, which are released upon the rupture of the host cell membrane and infect neighboring cells or enter the bloodstream. Once the trypomastigotes reach the bloodstream, the parasite circumvents complement-mediated lysis and opsonization with the aid of surface proteins, such as calreticulin and GP160 (17, 18). These proteins disrupt the initial attachment of mediators from the classical, alternative, and lectin complement pathways and dismantle the C3 convertase, a key step in all three pathways (19, 20). Thus, the parasite is allowed to disseminate through the bloodstream to many tissues during the acute phase. *T. cruzi* uses several other strategies to delay the generation of an effective immune response. During the initial phase of infection, the parasite elicits polyclonal B cell activation and hypergammaglobulinemia based on parasite-derived B cell mitogens. The antibodies produced by these cells are not parasite specific and are inefficient in controlling infection (21, 22). With the stimulation of innate immune receptors, such as the intracellular toll-like receptors (TLRs) 7 and 9, followed by proinflammatory cytokine production, a Th1-focused immune response is established (23–25). This response leads to the production of *T. cruzi*-specific CD8^+^ cells directed to parasite immunodominant epitopes, derived from the *trans*-sialidase family, that are important for controlling parasitemia and tissue parasitism (26, 27). However, this focused immune response fails to clear parasite infection, leading to the chronic phase of Chagas disease. This control is not detrimental to *T. cruzi*, as it reduces host mortality while maintaining parasite infectivity toward its reduviid insect vectors. In this review, we will focus on the major processes behind the parasite’s survival during the acute phase of Chagas disease.

## The Initial Phase of Infection: *T. cruzi* Invasion of Non-Professional Phagocytic Cells

Once a metacyclic trypomastigote penetrates the host through mucosa or lesions in the skin, it encounters host tissue cells and immune cells that populate or are recruited to that tissue. Poor parasite migration to surrounding tissues or draining lymph nodes and the evidence of parasite proliferation at the site of infection suggest that, immediately after the initial infection, the parasite invades tissues, rather than immune cells (28). In fact, in addition to being passively internalized by phagocytic cells, *T. cruzi* has the ability to invade any nucleated host cell.

*Trypanosoma cruzi* can actively invade a wide range of non-professional phagocytic cells through two different mechanisms. The first strategy that occurs in 20–30% of the cases is through a lysosome-dependent route, which induces Ca^2+^ signaling by inositol triphosphate (IP_3_) generation, followed by the recruitment and fusion of host cell lysosomes at the parasite entry site (29–33). The second pathway, which occurs in 70–80% of invasions, is via invagination of the plasma membrane, followed by intracellular fusion with lysosomes (32, 33). Regardless of the entry route, lysosomal fusion is essential for retaining the highly mobile trypomastigotes inside the host cell; otherwise, the parasite escapes to the extracellular environment and, therefore, does not establish a productive infection (33, 34). Additionally, the lysosomal acidification of the parasitophorous vacuole contributes to trypomastigote-to-amastigote differentiation that takes place in the cytoplasm. After a transient, but crucial, association of the trypomastigotes with the lysosome-like parasitophorous vacuole, also known as a phagolysosome, this structure is disintegrated by the parasite through the action of a low pH-dependent pore-forming protein (35, 36). This process is mediated by the desialylation of the phagolysosome membrane. The lysosome-like parasitophorous vacuole internal surface is coated with two major proteins that are greatly sialylated, known as lysosome-associated membrane proteins 1 and 2 (LAMP 1 and 2) (37–39). The presence of sialic acid residues appears to protect the parasitophorous vacuole membrane from lysis. In fact, trypomastigotes escape earlier from the phagolysosome in sialic acid-deficient Lec 2 cells than from wild-type cells (38, 40). In the acidic environment of the phagolysosome, however, the parasite surface protein *trans*-sialidase is shed and becomes active due to the low pH. Active *trans*-sialidase then transfers the sialic acid from LAMP proteins to parasite surface protein mucins, and this desialylation of the LAMP proteins renders the phagolysosomal membranes more susceptible to rupture (38, 39). Once the phagolysosome is destroyed and the trypomastigote reaches the cytoplasm, it differentiates into the replicative amastigote form and, after several rounds of replication, amastigotes differentiate into the bloodstream-infective trypomastigotes. These highly motile forms cause the rupture of the host cell membrane and can either infect neighboring cells or reach the bloodstream to disseminate the infection to distant tissues.

### Role of Host-Derived Nitroxidative Stress in *T. cruzi* Infection

**Figure 1:**
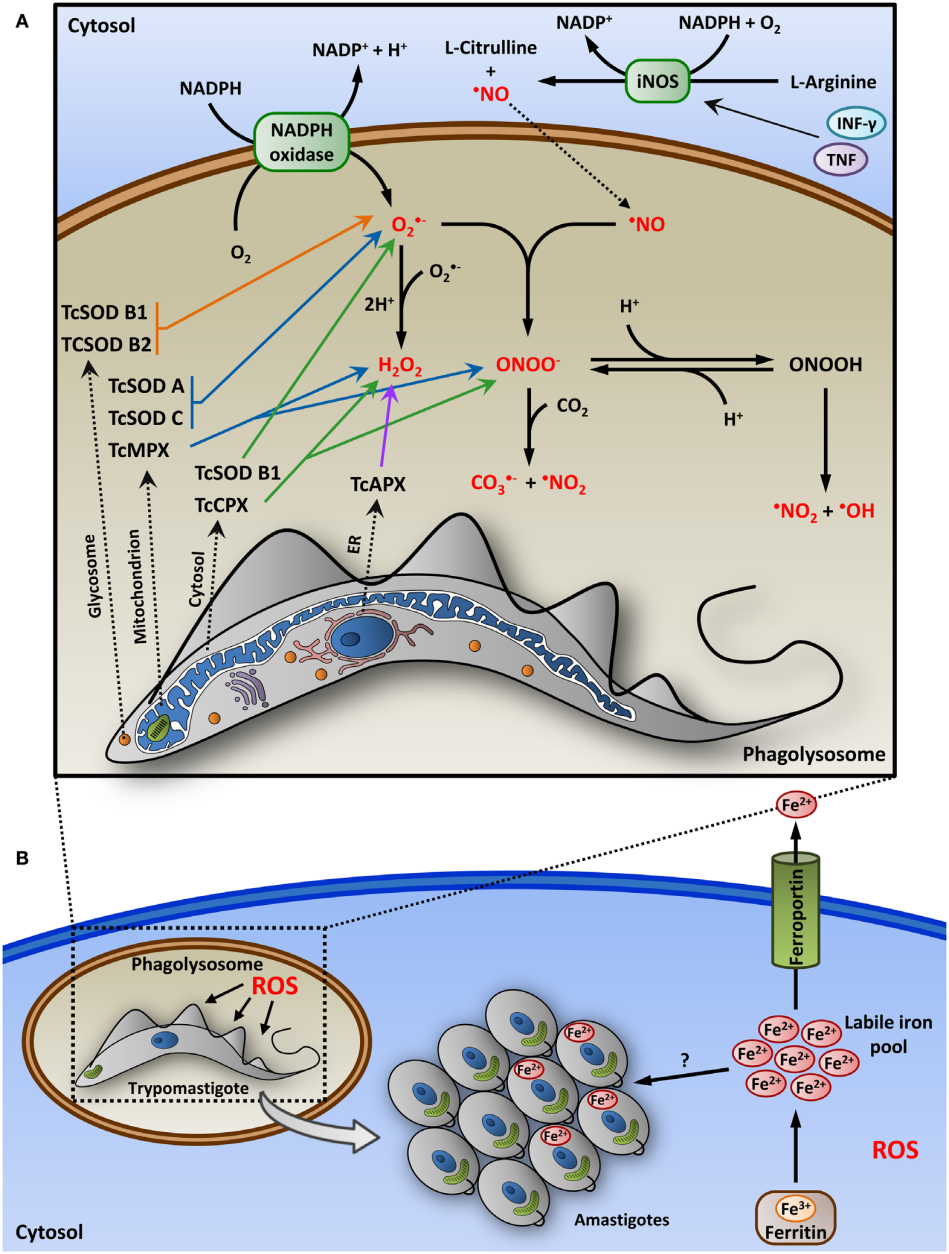
**Role of host-derived nitroxidative stress in *T. cruzi* infection**. **(A)** After the phagocytosis of the parasite, macrophage membrane-associated NADPH oxidase is activated, producing the superoxide radical $\left({\text{O}}_{2}^{•-}\right)$ that can be converted into H_2_O_2_ inside the lumen of the phagolysosome. Macrophages stimulated with proinflammatory cytokines (IFN-γ and TNF) induce the expression of nitric oxide synthase (iNOS), generating nitric oxide (^•^NO) in the cytoplasm from the oxidation of l-arginine. ^•^NO then diffuse into the phagolysosome vacuole and react with ${\text{O}}_{2}^{•-}$ to form peroxynitrite (ONOO^−^), a potent oxidant. Secondary free radicals, such as carbonate $\left({\text{CO}}_{3}^{•-}\right)$, nitrogen dioxide (^•^NO_2_), and hydroxyl (^•^OH) radicals, are produced from ONOO^−^. These reactive oxygen species (ROS, indicated in red) can cause various cellular damages and parasite death within the phagolysosome. To survive in this highly oxidative environment, the parasite has a complex network of antioxidant enzymes, as peroxidases (TcAPX, TcCPX, and TcMPX) and superoxide dismutases (SOD), which act in the detoxification of ROS, and are distributed in various cellular compartments, such as glycosomes, mitochondrion, cytosol, and endoplasmic reticulum (ER). Enzymes derived from glycosome, mitochondrion, cytosol, and ER are indicated by orange, blue, green, and purple arrows, respectively. **(B)** To establish a productive infection, trypomastigotes should escape from the phagolysosome to the cytosol, where it differentiates into replicative amastigotes. In the cytosol of macrophages, ROS, instead of being detrimental to the parasite, can promote the intracellular growth of *T. cruzi* by a mechanism that may involve facilitating amastigote access to iron. In the cytosol, iron can be stored as ferric iron (Fe^3+^), a redox-inert form, associated with ferritin or can be exported from the cell as ferrous iron (Fe^2+^) through ferroportin, a macrophage-specific iron exporter. The expression of ferroportin and ferritin is upregulated by antioxidants, which can lead to reduced levels of labile iron pool in the cytosol. The mechanism of iron uptake by amastigotes is unknown, but the parasite may be dependent on the intracellular labile iron pool for growth.

*Trypanosoma cruzi* can also be passively internalized by phagocytic cells. Resident macrophages at the site of infection are among the first professional phagocytes to be invaded by the parasite (41, 42). To establish a productive infection in macrophages, *T. cruzi* must endure the extremely oxidative environment inside the phagolysosome (43). To this end, *T. cruzi* has a complex network of antioxidant enzymes, such as peroxidases and SODs, that protect the parasite against macrophage-released reactive oxygen and nitrogen species (44) (Figure 1).

During phagocytosis, the trypomastigotes trigger activation of a macrophage membrane-associated NADPH oxidase, resulting in the continuous production of superoxide radical anions $\left({\text{O}}_{2}^{•-}\right)$, which can be converted to H_2_O_2_ by SOD (43, 45, 46). During *T. cruzi* infection and Chagas disease progression, reactive oxygen species (ROS; e.g., ${\text{O}}_{2}^{•-}$, H_2_O_2_, and ^•^OH) can be generated as a consequence of immune-mediated cytotoxic reactions, secondary damage to mitochondrion, and tissue destruction caused by the parasite. Thereafter, ROS can oxidize DNA, proteins, and lipids, killing the parasite (47).

Proinflammatory cytokines (IFN-γ and TNF) triggered by *T. cruzi* acute infection also stimulate infected macrophages to produce large amounts of nitric oxide (^•^NO) via the enzymatic activity of inducible nitric oxide synthase (iNOS), which oxidizes l-arginine and transfers electrons from NADPH (47–49). ^•^NO affects parasite survival in the macrophage by chemically modifying cysteine-containing proteins, inhibiting the catalytic activity of cruzipain, and binding to parasite metalloproteins (49, 50).

Once generated, ^•^NO can react with ${\text{O}}_{2}^{•-}$ to produce peroxynitrite (ONOO^−^), a potent oxidant and cytotoxic molecule that is highly effective against *T. cruzi* (46, 51). Peroxynitrite can damage cells directly by lipid peroxidation (harming membrane integrity and membrane protein function), as well as mitochondrial function and may result in apoptotic or necrotic cell death (46, 47). Moreover, secondary intermediate free radicals produced from ONOO^−^, such as hydroxyl (^•^OH), nitrogen dioxide (^•^NO_2_), and carbonate $\left({\text{CO}}_{3}^{•-}\right)$ radicals, can participate in the oxidation and nitration of proteins, lipids, and DNA, leading to mutations and transcription errors (43, 46). The oxidative stress caused by ^•^NO production can also be detrimental to the host, due to its high tissue-damaging potential (49). In fact, it has been shown that continuous exposure to nitroxidative-stress-induced damage can lead to Chagas disease progression and the development of myocarditis (47).

The parasite antioxidant network consists of various enzymes and non-enzymatic molecules distributed in diverse cellular compartments: the cytosol, ER, mitochondrion, and glycosome (Figure 1A). The final electron donor for all the enzymatic systems is the NADPH, which is derived from the pentose phosphate pathway, and their reducing equivalents are delivered to enzymatic detoxification systems via dithiol trypanothione T(SH)_2_ and the thioredoxin homolog tryparedoxin (TXN) (43). T(SH)_2_ is synthesized from two molecules of glutathione (GSH) and one spermidine by the enzyme trypanothione synthetase (TcTS) (52).

*Trypanosoma cruzi* has five peroxidases (also called peroxiredoxins) operating in its peroxide detoxification system (16). Cytosolic tryparedoxin peroxidase (TcCPX) and mitochondrial tryparedoxin peroxidase (TcMPX) have the ability to detoxify endogenous and macrophage-derived peroxynitrite, H_2_O_2_, and small-chain organic hydroperoxides (16, 53). Ascorbate-dependent heme-peroxidase (TcAPX), present in the ER, confers resistance against H_2_O_2_ (16, 54). Glutathione peroxidase-I (TcGPXI, located in the glycosome and cytosol) and glutathione peroxidase-II (TcGPXII, situated in the ER) confer resistance against lipid- and hydroperoxides (16, 55, 56).

Additionally, *T. cruzi* contains four iron SODs, which protect the parasite from the direct cytotoxic effects of ${\text{O}}_{2}^{•-}$ and, hence, inhibit the formation of ONOO^−^ by superoxide radical detoxification. TcSODs A and C neutralize the ${\text{O}}_{2}^{•-}$ produced in the mitochondrion, TcSOD B1 acts in the cytosol, and TcSOD B1-2 acts in the glycosomes (43, 57).

Several studies have described the role of *T. cruzi* antioxidant enzymes as virulence factors (43). The overexpression of the peroxiredoxins TcCPX and TcMPX in *T. cruzi* results in cell lines that readily detoxify ROS generated *in vitro* or released by activated macrophages (16, 51). The protective effects of peroxidase TcCPX have also been observed *in vivo*; when compared to mice infected with wild-type parasites, mice infected with TcCPX-overexpressing *T. cruzi* showed increased parasitemia and higher inflammatory infiltrates in the skeletal muscle and heart (51). Parasites overexpressing TcAPX were more resistant to H_2_O_2_ but were not resistant to peroxynitrite (16). Proteomic analyses have suggested the upregulation of the *T. cruzi* antioxidant network members TXN, TcTS, TcAPX, TcMPX, and TcSOD A in the infective metacyclic trypomastigote when compared with the non-infective epimastigote stage, reinforcing the role of these enzymes in *T. cruzi* survival inside the mammalian host (58, 59). Peroxiredoxins (TcCPX and TcMPX) and a trypanothione reductase (TcTR) were upregulated during the metacyclogenesis process regardless of the *T. cruzi* strain, as observed after an analysis of 10 different isolates (44). Peroxidases were also observed in increased levels in the metacyclic forms of these virulent strains compared with attenuated isolates (44). These studies highlight the importance of the parasite antioxidant enzyme network in the successful establishment of host infection.

Reactive oxygen species are labile molecules and many of their effects are due to their rapid accumulation in different cellular compartments, such as macrophage phagolysosome. During *T. cruzi* infection, large amounts of ${\text{O}}_{2}^{•-}$ are generated inside the phagolysosome after phagocytic stimulus (60). This radical is maintained for only 90–120 min and presents a limited diffusion capacity through the membrane due to its anionic nature (61). Although synthesized in the cytoplasm, ^•^NO is diffused into the phagolysosome vacuole due to its hydrophobic properties (60) and has a half-life of approximately 24 h (61). In the phagolysosome, ^•^NO reacts with ${\text{O}}_{2}^{•-}$ generating ONOO^−^, which presents a short half-life and high diffusion capacity (60). Parasite survival within the phagolysosome is broadly affected by macrophage production of ONOO^−^ during the first hours of infection (60).

Although the parasite faces an extremely oxidative environment inside the phagolysosome (Figure 1A), trypomastigotes are associated with this compartment transiently and after 24 h post-infection escape to the cytosol where the parasite remains as replicative amastigotes during the majority of its intracellular life cycle. A recent study has demonstrated an unexpected role of the oxidative stress in promoting *T. cruzi* infection. Paiva et al. (62) have shown that once the parasite reaches the cytosol of macrophages, oxidative stress can also contribute to parasite burden by a mechanism that may involve facilitating amastigote access to iron, which is critical for parasite growth (Figure 1B). Peritoneal macrophages from mice infected with the *T. cruzi* Y strain treated with cobalt protoporphyrin (CoPP) (an activator of the transcription factor NRF2, which orchestrates antioxidant responses), as other antioxidants, lead to a notably reduced parasite burden (62). It has also been demonstrated that pro-oxidants promote *T. cruzi* growth and reverse the host-protective effects of CoPP (62). Similar results were observed *in vivo*, where CoPP reduced parasitemia and tissue parasitism in infected mice (62). The protective effect of CoPP in *T. cruzi* infection is independent of T cell-mediated immunity and does not involve apoptotic clearance of infected cells or effectors that act against the parasite, such as type I IFN, TNF, or ^•^NO (62). These results suggest that the deleterious effects of antioxidants on parasite may occur by a mechanism different from classical innate or adaptive immune responses. Paiva et al. (62) demonstrated that the sequestration of iron, present in the host cytoplasm, is most likely involved in the parasite burden-reducing effects mediated by antioxidants, once the labile iron pool is reduced by the treatment of infected cells with antioxidants. Interestingly, these authors observed that induction of antioxidant responses reduced the parasite load in macrophages, but not in other cell types (62), suggesting that this may be a macrophage-specific mechanism. This can be explained by the role of macrophages as iron storage *in vivo*. Intracellularly, iron can be used in metabolic pathways in its ferrous form, which can also catalyze the formation of free radicals and, therefore, its concentration in the cytosol has to be tightly regulated. To this end, iron can be stored in the cytosol as ferric iron, a redox-inert form, associated with ferritin. Ferrous form can also be exported from the cell through ferroportin, a macrophage-specific iron exporter (63). The expression of ferroportin and ferritin is upregulated by the antioxidant response regulator NRF2 (64, 65), which can lead to reduced levels of labile iron pool in the cytosol. The mechanism of iron uptake by amastigotes is unknown, but the parasite may be dependent on the intracellular labile iron pool for growth (Figure 1B). This pathway could be the basis for the unexpected effect of antioxidants in reducing *T. cruzi* infection. Contrasting results were, however, observed in other studies, in which antioxidants had no impact in *T. cruzi* CL Brener infection (51) or increased the parasite burden in mice infected with strain Sylvio X10/4 (66). This latter study did not evaluate macrophage parasitism and, therefore, ROS production may be required to control parasitism in particular tissues (62). Additionally, strain-specific factors, such as level of expression of antioxidant enzymes, kinetics of association with the phagolysosome, and iron uptake efficiency, may contribute to differential resistance/susceptibility of distinct *T. cruzi* strains to the oxidative environment and outcome of the infection.

## Pattern-Recognition Receptors and Innate Immunity Against *T. cruzi*

Pattern-recognition receptors (PRRs) have been described as one of the first line of immune defense against various pathogens, including protozoans (67, 68). PPRs are expressed by cells of the innate immune system and are responsible for the recognition of molecules that are broadly shared by pathogens but distinguishable from host molecules, collectively referred to as pathogen-associated molecular patterns (PAMPs). TLRs are among of the best-characterized PPRs and detect PAMPs that are either located on the cell surface or in the lumen of intracellular vesicles, such as endosomes or lysosomes. These receptors are more abundant in antigen-presenting cells, such as macrophages and dendritic cells, but have also been described in T cells and some somatic cells (68–71). TLR activation leads to the production of proinflammatory cytokines and chemokines that in turn lead to the recruitment of phagocytic cells to the infected tissue, which are important not only for initial infection control but also for molding the subsequent adaptive immune response (25, 68, 72). A total of 12 and 10 TLR family members have been identified in mice and humans, respectively. TLRs 1–9 are shared between mice and humans, whereas TLR11, TLR12, and TLR13 are restricted to mice, and TLR10 is expressed only in humans (73, 74). Some TLRs function as homodimers, such as TLR4 and TLR9, whereas others are heterodimers, such as TLR2/6. After stimulation, these receptors undergo required conformational changes to recruit TIR-domain-containing adaptor molecules, which, with the exception of TLR3, lead to a MyD88-dependent signaling cascade that culminates in the production of proinflammatory cytokines (25, 68, 69).

**Figure 2:**
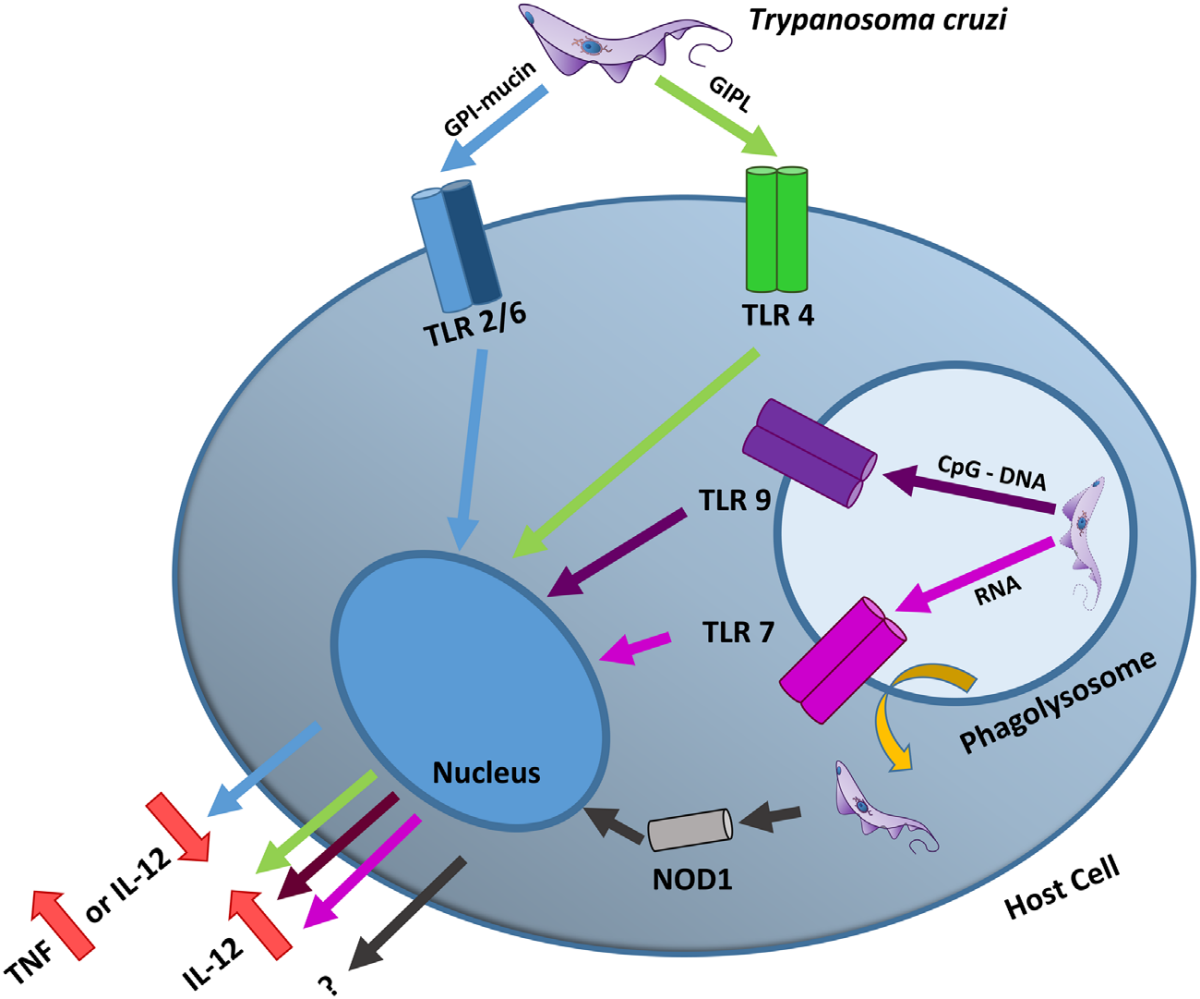
***T. cruzi* TLR and NLR activation**. *T. cruzi* possesses several molecules capable of stimulating TLRs. The activation of the heterodimer TLR2/6 by parasite GPI-mucins can lead to TNF production in macrophages or to the inhibition of IL-12 in dendritic cells (blue arrows). By contrast, the activation of TLR4 by parasite GIPLs (green arrows), TLR9 by parasite CpG DNA motifs (purple arrow) and TLR7 by parasite RNA (pink arrow) all result in the production of proinflammatory cytokines, such as IL-12. After the parasite escapes from the phagolysosome, it can activate the cytoplasmic NOD1 receptor. Although this receptor is important for controlling the infection, its mechanism of action is still unknown.

Toll-like receptors have a critical role in host resistance to *T. cruzi* infection, as evinced by a remarkable increase in the susceptibility of MyD88-deficient mice infected with *T. cruzi* compared with that of WT mice. This higher susceptibility is associated with the impaired production of IL-12 and IFN-γ proinflammatory cytokines, which are important for driving the Th1-directed protective immune response (75). *T. cruzi* has several molecules that can strongly stimulate TLRs, such as the surface molecules mucin and glycoinositolphospholipid (GIPL), as well as parasite DNA and RNA sequences (24, 25, 76–78) (Figure 2).

Mucins are GPI-anchored surface proteins that coat the entire surface of the parasite and are enrolled in immune evasion and host cell adhesion/infection processes (79, 80). The *T. cruzi* trypomastigote mucin GPI anchors, especially the unsaturated fatty acid at the sn-2 position, are potent stimulators of the extracellular heterodimer TLR2/6. *In vitro* stimulation of TLR2/6 by *T. cruzi* GPI-mucins leads to the production of proinflammatory cytokines, such as IL-12 and TNF, as well as nitric oxide, which are related to a Th1-focused immune response that is important to control parasitemia and tissue parasitism (67, 74, 81). However, in contrast to *in vitro* experiments, *in vivo* assays showed that TLR2-deficient mice infected with *T. cruzi* develop a strong proinflammatory immune response with higher IFN-γ serum levels than those of WT mice, suggesting an immunoregulatory role for TLR2 during *T. cruzi* infection (74, 75). Recently, Gravina and coworkers suggested that TLR2 assumes different functions depending on the host cell type, acting as a TNF producer in macrophages and as an immunoregulator in dendritic cells (78). *T. cruzi* covers its whole surface with as many as 2 × 10^6^ mucin molecules (79, 80); the abundance of this molecule may be important for stimulating dendritic cells in a TLR2-dependent manner during the initial steps of infection, leading to an immunoregulatory effect *in vivo*, and may contribute to the delayed immune response and antibody production against the parasite (78, 82).

Glycoinositolphospholipids are free GPI anchors present in all *T. cruzi* life stages (72, 83–85). *T. cruzi* GIPLs share a core conserved structure [Manα(1 → 2) Manα(1 → 2) Manα(1 → 6) Manα(1 → 4) GlcNα(1 → 6) *myo*-inositol 1-PO_4_-lipid], in all parasite stages and among different strains, although considerable variability exists in both the lipid and glycan portions of these molecules (84, 86). GIPLs from G, Y, and Tulahuén strains contain ceramide, whereas those from CL contain alkylacylglycerol and dihydroceramide (83, 84, 86, 87). Lipid remodeling has also been detected in GPI-anchored proteins and GIPLs in different forms of *T. cruzi* (79, 87–90). This distinct composition confers distinct biological functions, as low concentrations of GIPLs containing ceramide have been shown to induce apoptosis and regulate the activity of macrophages and dendritic cells (91, 92). This difference is also important in TLR recognition, as GIPLs containing ceramide are recognized by the homodimer TLR4, while GIPLs containing alkylacylglycerol are agonists of TLR2/6 (74, 76, 93). Although TLR2/6 stimulation by GPI-mucin appears to be 100-fold more efficient in stimulating the immune response *in vitro* (74, 93), this stimulation has also been associated with immunoregulation (74, 75, 78), whereas an anti-inflammatory outcome with respect to TLR4 stimulation has not yet been described.

In contrast to TLR2/6 and TLR4, which are localized on the cell surface, TLR7 and TLR9 are expressed in the ER and, upon *T. cruzi* cell invasion, are translocated to endolysosomes, where they recognize immunostimulatory motifs derived from parasite RNA or DNA, respectively (23–25, 94, 95). As *T. cruzi* invades the host cell and reaches the phagolysosome environment, nucleic acid molecules from lysed parasites stimulate TLR7 and 9, leading to the production of Th1 proinflammatory cytokines important for controlling the infection (24, 77, 95, 96). The immunostimulatory CpG DNA motifs are not randomly distributed in the parasite genome; instead, they are enriched in genomic regions that encode large gene families of surface proteins, such as mucins, *trans*-sialidases, and mucin-associated surface proteins (MASPs) (24). As most of the proteins encoded by these genes are involved in parasite immune evasion mechanisms or host cell adhesion/invasion (14, 15, 80), there appears to be a trade-off between the need to invade cells and CpG immune stimulation via TLR9. One of the mechanisms that may reduce this drawback is the ability of *T. cruzi* to escape from the phagolysosome, reducing the chance of lysis and, therefore, minimizing TLR9 activation. Concomitantly, the immunoregulatory effect of TLR2 stimulation by GPI-mucin in dendritic cells may also balance TLR9 and TLR7 activation by parasite DNA and RNA (78), respectively, at least in the initial phases of infection.

In addition to TLR, other innate immune receptors important in controlling *T. cruzi* infection are the nucleotide-binding oligomerization domain (Nod)-like receptors (NLR). NLRs are localized in the cytoplasm or are associated with the plasma membrane of mammalian cells. NLRs are related to MAP kinase and NF-κB activation (NOD1 and NOD2) or with the production of a caspase 1-dependent inflammasome (NLRP3) (97). *In vitro* studies have shown that although macrophages from NOD1^−/−^ and NOD2^−/−^ mice infected with *T. cruzi* failed to produce nitric oxide (^•^NO) when stimulated with IFN-γ, only NOD1^−/−^ mice failed to eliminate the intracellular parasites (98). NOD1^−/−^ mice infected with *T. cruzi* showed threefold higher parasitemia than WT and NOD2^−/−^ mice, and succumbed 24 days post-infection (98). Although NOD1 receptors appear to be important for *T. cruzi* infection control, the mechanisms involved are still unknown, as a deficiency in this receptor does not impair cytokine production *in vivo*, and *T. cruzi* lacks any previously described NOD1 agonists (98).

## Complement Evasion

**Figure 3:**
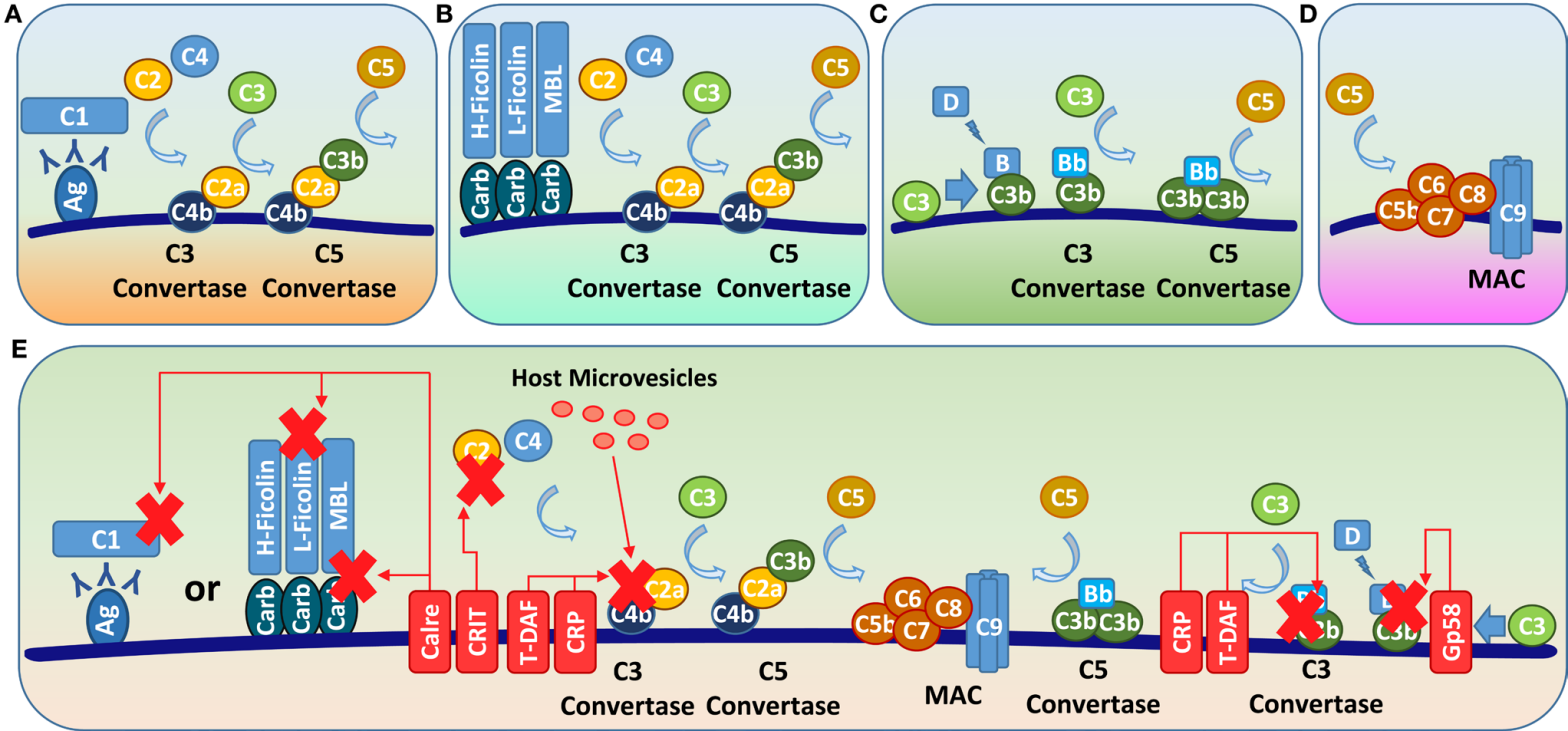
***T. cruzi* complement evasion mechanisms**. There are three complement pathways: classic, alternative, and lectin. **(A)** In the classical pathway, antibodies bound to pathogen antigens interact with the complement C1 protein, which cleaves C2 and C4 to generate C2a and C4b; these molecules bind to the pathogen surface to form the C3 convertase C4b2a. **(B)** In the lectin pathway, MBL or ficolin binds to mannan or glycosylated molecules, respectively, on the pathogen surface, and cysteine proteases bound to these molecules cleave C2 and C4, also generating the C3 convertase C4b2a. **(C)** In the alternative pathway, spontaneously hydrolyzed C3b or C3b originating from the other complement pathways interacts with factor B, which is cleaved into Bb by factor D, forming the C3 convertase C3bBb. The C3 convertases from all the complement pathways interact with newly cleaved C3b, forming a C5 convertase that cleaves C5 into C5b. **(D)** C5b interacts with C6–C9 to form the MAC, leading to the pathogen lysis. **(E)** To avoid lysis, *T. cruzi* relies on molecules, such as calreticulin and gp58/68 (Gp58), which block the initial steps of classic/lectin or alternative pathways, respectively, and CRIT, T-DAF, CRP, and host-derived microvesicles that disrupt or block C3 convertase assembly. Ag, antigen; Carb, carbohydrate; Calre, calreticulin.

After the first round of intracellular replication and host cell rupture, *T. cruzi* reaches the mammalian bloodstream and becomes a target of the complement pathways. The complement system consists of soluble proteins that interact with pathogen structures and activate a cascade of proteases that eliminate invading microorganisms. There are three complement pathways: classical, alternative, and lectin (Figures 3A–C). Although these pathways differ in the initial steps of their respective cascades, all three converge to produce a C3 convertase and then a C5 convertase, leading to the formation of the membrane attack complex (MAC) and subsequent pathogen lysis (Figure 3D).

*Trypanosoma cruzi* initially becomes a target of the host alternative and lectin complement pathways. The lectin pathway is activated by the binding of mannan-binding lectins (MBLs) or ficolins to the mannan or carbohydrates of the parasite surface, respectively (99) (Figure 3B). This pathway is responsible for almost 70% of parasite complement-mediated lysis during infection (20, 99, 100). The alternative pathway is activated either by a low rate of spontaneous conversion of C3 to C3b or by C3b generated from the other complement pathways (100) (Figure 3C). As the infection progresses and anti-*T. cruzi* antibodies are produced, antibodies bound to parasite surface molecules interact with the complement C1 molecule, activating the classical pathway (Figure 3A).

To escape complement-mediated lysis, *T. cruzi* relies on a large set of molecules that act by blocking different steps of the complement pathways (20, 101) (Figure 3E). *T. cruzi* impairs the lectin pathway via calreticulin, a 45-kDa surface molecule (102) that binds to host MBL collagenous tails, preventing their interaction with parasite mannan (18), and also interacts with l-ficolin, preventing C4–C4b conversion (103). As anti-*T. cruzi* antibodies are produced, calreticulin also interacts with C1, preventing its interaction with C4 and inhibiting the classical complement pathway (18, 104, 105). Therefore, calreticulin is able to disrupt the initial steps of both the classical and lectin complement pathways, and, because it reduces the formation of C3 convertase, calreticulin also indirectly inhibits the alternative pathway.

Complement regulatory protein (CRP), also called GP160, is a trypomastigote GPI-anchored surface protein that binds to C3b and C4b, dissociating the classical and alternative complement C3 convertase (17, 106). Beucher and Norris have described several CRP paralogs within the *T. cruzi* genome that share sequence similarity with *T. cruzi trans-*sialidase superfamily members lacking TS activity (107). Nevertheless, the involvement of these sequence-related CRP paralogs in blocking the activation of the alternative and classical pathways needs experimental validation.

Complement C2 receptor inhibition trispanning (CRIT) is a *T. cruzi* transmembrane protein that blocks C2 cleavage into C2a, preventing the lectin and classical complement pathway-mediated formation of C3 convertase (99, 108). Trypomastigote decay-accelerating factor (T-DAF) is an 87- to 93-kDa protein with similarity to human decay-accelerating factor (DAF), which interferes with C3 convertase assembly efficiency, potentially affecting the three complement pathways (19, 109). *T. cruzi* gp58/68 also inhibits C3 convertase assembly, but only in the complement alternative pathway, by preventing the binding of factor B to surface-fixed C3b (110).

Finally, it has recently shown that *T. cruzi* induces the release of plasma membrane-derived vesicles from host cells (111). These vesicles are involved in diverse immune evasion processes, including binding to and inhibiting the activity of the complement C3 convertase C4b2a (111), and will be further discussed in the microvesicles section of this review.

In summary, *T. cruzi* complement evasion focuses on diverse molecules that disrupt or inhibit C3 convertase formation, a key step in all complement pathways, or neutralizes the initial steps of the complement cascade (Figure 3). As all complement pathways converge with C3 convertase formation, disrupting this key step is an efficient way to disturb all complement-mediated responses simultaneously. In addition to being important in the complement cascade, C3b is also an opsonin, which is recognized by macrophages and induces phagocytosis (112). Therefore, inhibiting C3b formation may also reduce macrophage-derived parasite lysis during infection.

## The Role of Microvesicles in *T. cruzi* Infection

Microvesicles (MVs) are also known as microparticles, ectosomes, exosomes, or plasma membrane-derived vesicles (111, 113–115). MVs have a complex lipid bilayer structure and carry several cell-derived molecules, such as lipids, peptides, proteins, and nucleic acids (e.g., miRNAs and mRNAs), which can be transferred to and become functional in target cells (116–119). The release of plasma membrane-derived vesicles occurs at basal levels, but may be greatly increased by extracellular stimuli, such as parasitic infection (111, 120).

Recent studies have shown that MVs play an active role in intercellular communication inside an organism or between different organisms, as occurs during pathogen infections in a host (111, 115–118). Furthermore, the involvement of MVs in various diseases, such as thrombosis, cancer, pathogen infections, autoimmune diseases, and others, has also been observed (120). Vesicles may also participate in the delivery of pathogen virulence factors, contributing to the spread of the pathogen and successful immune evasion (111, 117, 118).

As discussed above, one of the first barriers encountered by parasites is the innate immune complement system. Recently, Cestari et al. (111) observed that *T. cruzi* induces the release of host plasma membrane-derived vesicles to evade innate immunity, by inhibiting complement-mediated lysis and also facilitating host cell invasion. At the beginning of the infection, metacyclic trypomastigotes induce MV release from blood cells, such as lymphocytes, monocytes, and macrophages, in a Ca^2+^-dependent process (111). The host-derived MVs predominantly inhibit the classical and lectin pathways of the complement system, increasing parasite survival. This inhibition is mediated by host MVs that bind to the C3 convertase C4b2a on the *T. cruzi* surface, leading to the inhibition of its catalytic activity (111).

Moreover, it has also been shown that lymphocytes- and monocytes-derived MVs carry the cytokine TGF-β, enhancing *T. cruzi* cell invasion and protecting the parasite from the complement-mediated lysis (111). This increase in cell invasion has also been demonstrated *in vivo*; mice infected with *T. cruzi* in the presence of MVs exhibited increased parasitemia (111).

In addition, parasite-shed vesicles may contain important virulence factors that contribute to the parasite–host interplay and the establishment of infection (117, 119). *T. cruzi*-derived MVs can act as messengers, preparing the cellular environment to facilitate infection, and thereby ensuring parasite survival (117, 121). This process occurs either through the interaction of parasite-derived MVs with host cell surface components or through the internalization of vesicles, which are accumulated in endocytic/phagocytic pathways (117). Proteomic analysis has revealed that the main components of the parasite-derived vesicles are TS/gp85 superfamily members, α-galactosyl-containing glycoconjugates, proteases, MASPs, and cytoskeleton proteins (117).

Previous inoculation with *T. cruzi*-derived MVs accelerates and enhances the mortality rate of infected mice, which develop more severe heart lesions with an increased number of intracellular amastigote nests (121). Furthermore, parasite vesicles induce IL-4 and IL-10 production in the heart and spleen and IL-10 and IL-12 production by resident peritoneal cells (121). Changes in host cell gene expression were also observed in HeLa cells upon the incorporation of parasite-derived extracellular vesicles containing tRNA-derived small RNAs (tsRNAs) from *T. cruzi* (119). The elicited response primarily modified the host cell extracellular matrix, cytoskeleton, and immune response pathways (119).

All together, these data indicate that both host- and parasite-derived plasma membrane MVs play an important role in the establishment and maintenance of parasite infection.

## Delayed Development of a Protective Immune Response: Polyclonal B Cell Activation, Smoke Screens, and Immunodominance

In contrast to other infectious pathogens that induce rapid changes in the gene expression of infected host cells (122), *T. cruzi* only exerts significant gene expression changes in human fibroblasts 24 h after infection (122). This delayed host transcriptional response coincides with the parasite escape from the phagolysosome to the cytoplasm and differentiation into the replicative amastigote forms. This sequence of events suggests that during the initial phase of a primary *T. cruzi* infection, the parasite does not trigger host PRRs, leading to silent entry (82, 122, 123). Besides the delayed changes in the gene expression of infected cells, *T. cruzi* immune activation coincides with the release of trypomastigotes from infected cells 4–5 days post-infection, suggesting that the parasite relies on mechanisms to avoid PAMP-derived immune stimulation during the first cycle of replication (28, 82). Three aspects may contribute to this silent entry: (i) the relatively slow kinetics of *T. cruzi* intracellular cycle, (ii) parasite escape from the phagolysosome, and (iii) immunoregulatory response meditated by TLR2/6 activation in dendritic cells. *T. cruzi* growth rate is significantly slower than virus and bacteria, taking longer to achieve a threshold necessary to mount a robust immune response, which is delayed to at least the end of the first round of intracellular replication (28). Also, parasite escape from the phagolysosome reduces its mortality, thus, reducing the amount of DNA and RNA immunostimulatory sequences available for TLR9 and TLR7 activation in this cellular compartment. Finally, the TLR2/6 immunoregulatory stimulation of dendritic cells by GPI-mucins could counteract other immune activation processes and could also delay the development of adaptive immune response (78). Another possibility for the immunologically silent entry is that *T. cruzi* PAMPs may not trigger an immunostimulatory response as effective as those of bacteria, since transgenic expression of bacterial PAMPs in *T. cruzi* enhanced the anti-parasite response leading to pathogen control and clearance (124).

After several rounds of infection/proliferation, a robust anti-*T. cruzi* immune response is developed, which is able to greatly reduce parasitemia and tissue parasitism. However, this immune response is unable to provide parasite clearance, as polymerase chain reaction (PCR) and immunocytochemistry assays have shown the presence of parasites in infected tissues in patients with cardiac (125–127) and digestive (128) manifestations. The delayed immune response and the inability to clear the parasite may be related to the large repertoire of highly polymorphic and immunogenic surface proteins that are coexpressed by the parasite (82, 123, 129, 130). This antigen arsenal may provide means of evading immune response that are distinct from the classic antigenic variation employed by parasites such as *Trypanosoma brucei* and *Giardia lamblia* (131–136).

Classic antigenic variation is achieved by the expression of identical antigenic variants on the surface of the majority of the cells in a parasite population while a small subset expresses different variants (131, 137–139). The immune response targets the parasites expressing the common variant while failing to identify those expressing rare variants (137). Long-term infection is achieved by varying the expressed antigens, leading to successive waves of parasitemia and clearance as novel antigenic determinants spread in the parasite population (133, 138, 139). There is no evidence that *T. cruzi* adopts this type of antigenic variation. Instead, the entire *T. cruzi* population simultaneously exposes a variety of antigenic surface proteins, such as mucins, *trans*-sialidase, and MASPs, encoded by highly polymorphic multigene families (22, 80, 82, 129, 130). The coexpression of this diverse antigenic repertoire drives the immune system into a series of spurious and non-neutralizing antibody responses, a mechanism known as a smoke screen, which delays the production of high-affinity anti-*T. cruzi* antibodies and the priming of effective T-CD8^+^ cells (22, 82, 140). The presence of a broad range of antigenic motifs may also be a mechanism to drive the antibody response away from catalytic sites of key parasite surface proteins. In fact, a strong humoral response against the *trans*-sialidases C-terminal repetitive motif shed acute phase antigen (SAPA) has been observed, followed by a weak antibody response against several epitopes at the N-terminal catalytic region in a later stage that was unable to inhibit the enzyme activity (141).

In addition to the high variability of parasite surface antigens, the presence of parasite-derived B cell mitogens also causes polyclonal B cell activation and hypergammaglobulinemia, resulting in a delayed parasite-specific antibody response (21, 22, 142, 143). This unfocused response is important for parasite survival, as most of the antibodies produced by splenic cells during the initial acute phase do not target the parasite, and specific anti-*T. cruzi* antibodies are only produced later (22). Interestingly, although the humoral response in the chronic stage shows a preferential IgG2a pattern, the acute infection comprises a broader range of immunoglobulin isotypes: IgM, IgG1, IgG2a, IgG2b, and IgG3 (22, 144). In addition to B cell mitogens, another driving factor of this polyclonal activation may be the coexpression and shedding of a large repertoire of immunogenic surface proteins, delaying the immune response to immunodominant epitopes. In fact, *trans-*sialidases and their terminal long tandem repeats have been shown to be T-independent polyclonal activators of mouse B cells (143, 145, 146). Even though polyclonal B-cell activation is transient and its role as a parasite escape mechanism needs further *in vivo* experimental validation, this may be a strategy that could contribute for parasite survival during the initial stage of infection, when parasitemia is low and the parasite has not yet reached the sites where it persists, such as muscle, adipose tissue, and nervous system.

In contrast to previous studies, Bryan and coworkers have shown that C57BL/6 mice infected with *T. cruzi* Y strain presented lower polyclonal B cell activation than BALB/c mice, suggesting that polyclonal activation is not a generalized response in *T. cruzi* infection and is highly dependent on the host strain (147). The authors associated this difference with the protective Th1-focused C57BL/6 immune response, in contrast to the susceptible Th2-focused response developed by BALB/c mice (147). Distinct parasite strains also show different degrees of B-cell polyclonal activation. Parasites from TcVI DTU such as CL (144) and Tulahuén (22) strains and the clone CL Brener (145) induced polyclonal B-cell activation in BALB/c and C3H/Hej mice, while polyclonal activation induced by Y strain was restricted to BALB/c (147). The TcVI DTU was originated by a hybridization event between TcII and TcIII strains (148), which result in an increased repertoire of multigene families encoding surface proteins when compared to Sylvio X10 (TcI) (149). As these surface proteins are highly immunogenic, this larger repertoire of antigens could contribute to B-cell polyclonal activation observed in infections by TcVI strains.

*Trypanosoma cruzi* antigens released in the intracellular host cell environment, either from live parasites shedding or parasite lysis, become available for presentation by the class I major compatibility complex (MHC) through the endogenous pathway (25, 26). This presentation promotes the priming of a strong but delayed CD8^+^ T immune response that is highly effective for controlling parasite levels, but only becomes evident 5–6 days post-infection, coinciding with the first round of intracellular replication (82, 150–153). The delayed anti-*T. cruzi* immune response may be due to the need for a sufficient number of antigen-producing amastigotes accumulating in the cytosol, and/or by the large number of different polymorphic antigens that are simultaneously expressed by the parasite. These antigens may compete for presentation through host cell MHC class I molecules, delaying a fast and focused immune response (82, 123, 152, 153). This immunologically silent initial phase of infection may allow the parasite to reach a critical level before activating the host immune system (82, 123, 154). As the infection advances, pathogen-specific T cells appear to preferentially recognize a small number of epitopes in a hierarchical manner, a process called immunodominance (26, 27, 152, 155). Immunodominant antigens can be selected based on several factors, such as the abundance of the parasite epitope and its affinity to MHC and T-cell receptors (26, 156–158). *Trans*-sialidases are among the major known CD8^+^ T immunodominant targets in *T. cruzi* infection, due to high expression in the infective forms and repetitive/antigenic content; as such, these enzymes have also been proposed as vaccine targets (27, 74, 152, 159–162). As previously stated, *trans*-sialidase is a highly polymorphic, multi-copy gene family in *T. cruzi*, with several potential immunogenic candidates that can generate an unfocused immune response. To overcome this, the anti-*trans*-sialidase immune response is focused on a relatively small number of epitopes encoded by multiple genes (82, 152). *Trans*-sialidase immunodominant antigens can account for more than 30% of the entire CD8^+^ response in mice (152) and a significant proportion in humans (153). The presence of subdominant/cryptic antigens was demonstrated after the tolerization of the major immunodominant epitopes of *T. cruzi* during infection in BALB/c and C57BL/6 mice (27). These mice exhibited an immune response against novel antigens and a transient increase in parasite load but were ultimately able to control the acute infection, suggesting that a focused immune response *per se*, but not the presence of these immunodominant antigens, is required to control the infection (27, 82). This result is not surprising, as the *trans*-sialidase gene family varies in sequence and expression among *T. cruzi* strains (148, 149, 163), and immunodominance also depends on interaction between the antigen and host receptors, which vary among host species. Although immunodominance is a well-described phenomenon in *T. cruzi*, its direct implications for parasite clearance are still under debate. While some authors state that specific T cells for a single epitope can hinder the development of immunity to several other epitopes, allowing a small set of variant parasites to escape from the immune system (26, 164), others argue that immunodominance is probably not the major factor governing *T. cruzi* escape from sterile immunity (27). The second group argued that vaccination to boost specific immunodominant epitopes enhanced mice protection, instead of being deleterious to the hosts by strongly focusing the immune response on the immunodominant epitope (161, 165), and the tolerization of immunodominant epitopes did not lead to higher parasite clearance (27).

## Conclusion

**Figure 4:**
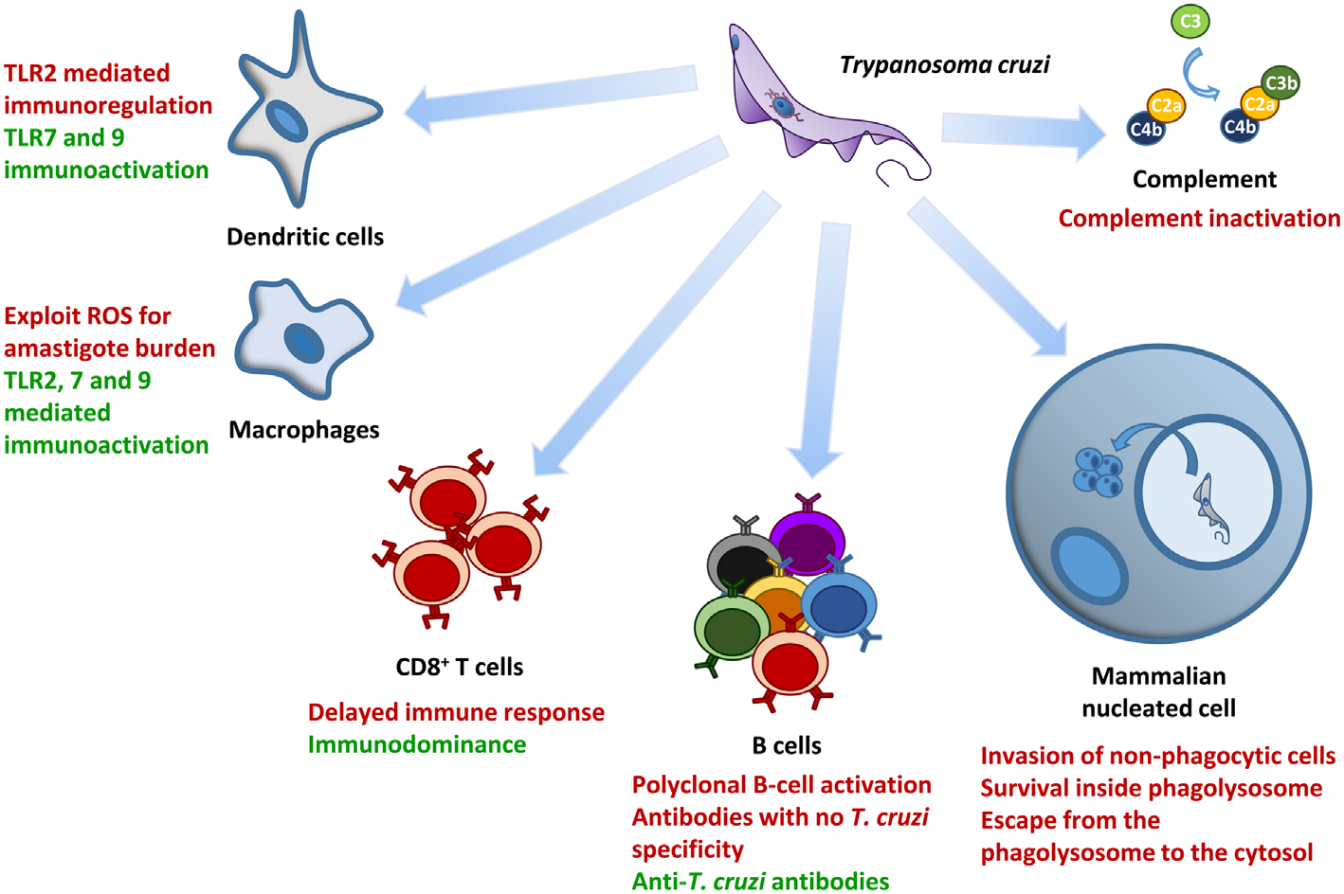
**Major mechanisms involved in *T. cruzi* survival and control during the initial phase of infection**. This figure summarizes the major interaction mechanisms between *T. cruzi* and several host components addressed in this review. Mechanisms associated with the control of parasite load are highlighted in green, whereas those involved with parasite modulation of the host immune system and/or with increased parasite load are highlighted in red.

*Trypanosoma cruzi* has been interacting and coevolving with humans for 6,000–9,000 years (5, 10, 166), and infecting wild mammals even longer (6–8, 167). Because of this extensive interaction with mammalian hosts and its obligatory parasitic lifestyle, this protozoan has developed several mechanisms to evade the host immune system (Figure 4), and simultaneously reduce host damage while maintaining its transmissibility to insect vectors (168). It is not surprising that as the disease reaches its chronic stage, only 30% of the patients progress to cardiac or digestive manifestations, whereas 70% show no clinical symptoms but are still able to infect triatomine insect vectors (168). However, when this equilibrium is lost and symptoms do occur, the disease causes great morbidity, resulting in a loss of 662,000 disability-adjusted life years (1–3) (WHO) (see text footnote 1). Among the trypanosomatids whose genomes have already been sequenced, *T. cruzi* exhibits the largest expansion of the multigene families that encode surface proteins, many of which are antigenic (130, 148, 163). A driving force for the expansion of these polymorphic surface proteins may be their involvement in the parasite’s ability to invade any mammalian nucleated cell, which is a critical strategy that allows the parasite to spread in different host tissues during the initial infection. In addition, this impressive surface protein polymorphism also contributes to antigenic variability, leading to the coexpression of several polymorphic antigens that delay the development of an effective immune response. The delayed immune activation in host cell newly infected with *T. cruzi*, the polyclonal B cell activation, and *T. cruzi* intra- and inter-strain surface antigenic variability makes prophylactic vaccine target identification nearly impossible (82). An effective pan-*T. cruzi* vaccine would have to include immunodominant and cryptic antigens from a broad variety of parasite isolates.

## Author Contributions

MC: participated in design and manuscript writing. JR-C: participated in design and manuscript writing. DB: participated in design, coordination, and manuscript writing.

## Conflict of Interest Statement

The authors declare that the research was conducted in the absence of any commercial or financial relationships that could be construed as a potential conflict of interest.

## Footnotes

^1^http://www.who.int/chagas/en/
